# Supplementary material for: Modulation of dopamine D1 receptors via histamine H3 receptors is a novel therapeutic target for Huntington's disease
Source: eLife. 2020 Jun 9;9:e51093. doi: 10.7554/eLife.51093 (PMC7282811; doi:10.7554/eLife.51093)
Supplement: Supplementary file 2. — Mouse striatal, cortical or hippocampal tissue were homogenized in 50 mM Tris-HCl buffer and ligand binding was performed with membrane suspension (see online methods). Binding parameters from saturation and competition curves were obtained using Grafit software by fitting the binding data to the equation previously deduced (equation (3) in Gracia et al., 2013. Data are mean ± SEM of experiments performed per triplicate (n = 6 HdhQ7/Q7 and n = 5 HdhQ7/Q111). [file elife-51093-supp2.docx]

Supplementary file 2

| **Receptor** | **BRAIN**  **REGION** | **Hdh^Q7 /Q7^**  **B_max_ (fmol/mg protein)** | **Hdh^Q7 /Q7^**  **K_D_ (nM)** | **Hdh^Q7/ Q111^**  **B_max_ (fmol/mg protein)** | **Hdh^Q7/ Q111^**  **K_D_ (nM)** |
| --- | --- | --- | --- | --- | --- |
|  | **Striatum** | 193 ± 6 | 0.27 ± 0.03 | 155 ± 6 | 0.26 ± 0.03 |
| **H_3_R** | **Cortex** | 146 ± 4 | 0.22 ± 0.02 | 152 ± 8 | 0.24 ± 0.04 |
|  | **Hippocampus** | 92 ± 1 | 0.20 ± 0.01 | 88 ± 3 | 0.37 ± 0.1 |
|  | **Striatum** | 1088 ± 50 | 0.17 ± 0.02 | 529 ± 32 | 0.11 ± 0.01 |
| **D_1_R** | **Cortex** | 99 ± 5 | 0.05 ± 0.01 | 73 ± 4 | 0. 01 ± 0.01 |
|  | **Hippocampus** | 257 ± 10 | 0.03 ± 0.02 | 187 ± 19 | 0.09 ± 0.07 |
