# Supplementary material for: Modulation of dopamine D1 receptors via histamine H3 receptors is a novel therapeutic target for Huntington's disease
Source: eLife. 2020 Jun 9;9:e51093. doi: 10.7554/eLife.51093 (PMC7282811; doi:10.7554/eLife.51093)
Supplement: Supplementary file 3. — RT-PCR was performed in striatal extracts from HdhQ7/Q7 and HdhQ7/Q111 at 4 and 8 months of age as described in materials and methods. Results were normalized to actin gene expression. Data represent mean ± SEM (n = 3–4) of experiments performed in duplicate and are expressed as fold change of wild-type animals. Student’s two-tailed t test was performed. [file elife-51093-supp3.docx]

Supplementary file 3

| **Receptor** | **BRAIN**  **REGION** | **Age** | **Hdh^Q7 /Q7^**  **mRNA fold increase** | **Hdh^Q7/ Q111^**  **mRNA fold increase** |
| --- | --- | --- | --- | --- |
| **H_3_R** | **Striatum** | 4 months | 1.01 ± 0.12 | 0.73 ± 0.15 |
|  |  | 8 months | 0.74 ± 0.18 | 0.87 ± 0.16 |
| **D_1_R** | **Striatum** | 4 months | 1.04 ± 0.17 | 0.83 ± 0.08 |
|  |  | 8 months | 0.82 ± 0.06 | 0.76 ± 0.07 |
